# Optimization Effect of Metakaolin on Macro- and Micro-Mechanical Properties of Composite Cementitious Materials under Different Curing Conditions

**DOI:** 10.3390/ma15238435

**Published:** 2022-11-26

**Authors:** Chao Liang, Yongming Xing, Xiaohu Hou

**Affiliations:** 1 College of Science, Inner Mongolia University of Technology, Hohhot 010051, China; 2 College of Civil Engineering, Inner Mongolia Technical College of Construction, Hohhot 010070, China; 3 College of Materials Science and Engineering, Inner Mongolia University of Technology, Hohhot 010051, China

**Keywords:** metakaolin-cement cementitious system, high-temperature steam curing, compressive strength, flexural strength, the micro-mechanical properties of C-(A)-S-H gel, Al/Si, ACI empirical model, copula function

## Abstract

To provide the theoretical basis for the engineering application of metakaolin as aluminum-rich pozzolanic ash materials, the promoting effect of metakaolin on the macro- and micro-mechanical properties of composite cement-based composite materials was explored under high-temperature steam and standard curing conditions. Analysis techniques, which involved thermogravimetric and nanoindentation coupled with scanning electron microscopy–energy dispersive X-ray spectroscopy, were used. To analyze the experimental data, the ACI empirical formula and the copula function were used. The correlation among the hydration degree of the MK-cement cementation system, the Al/Si of the C-(A)-S-H phase, and the nanomechanical properties of the C-(A)-S-H gel phase was investigated. According to prior research, the macroscopic mechanical properties and the substitution rate of the MK-cement composite cementitious system can be improved under high-temperature curing conditions in a short period, in which the optimum substitution rate of metakaolin is 20%. The ACI empirical formula was used to demonstrate the functional relationship between the metakaolin replacement rate, curing time, and compressive strength. The MK-cement cementation system can eliminate the defect phase, reduce the CH phase content, and then increase the C-(A)-S-H gel phase content and bulk density. The micro-mechanical properties of the C-(A)-S-H gel phase rises due to its phase content and Al/Si ratio. Furthermore, the copula function verifies the dependence of the nanomechanical properties of C-(A)-S-H gel and Al/Si.

## 1. Introduction

The interest in the development of cement substitute materials, such as fly ash, slag, and metakaolin (MK), has increased due to the growth of the building industry, stricter performance standards placed upon materials, and the higher sustainability criteria applied to precast and mass concrete projects, such as prefabricated constructions and integrated urban plumbing corridors [1,2]. Aluminum-rich pozzolan material is a suitable alternative for the partial or complete substitution of Portland cement in the manufacturing of concrete at comparable performance and cost. According to previous studies, aluminum-rich pozzolan materials demonstrate excellent pozzolanic and filling effects [3,4]. They react with the portlandite (CH) of cement in the pozzolanic reaction to produce a secondary C-(A)-S-H gel [5]. The newly formed hydration products tend to physically fill the void between the larger particles, which are occupied by water and unhydrated particles, reducing the porosity of the matrix [6,7]. Furthermore, a portion of the new C-(A)-S-H gel acts as seeds, providing nucleation sites for cement hydration products, resulting in compactness and grain refinement of the matrix [8].

Furthermore, aluminum-rich pozzolan material helps in improving the silicon chain structure. The Si ions in the MK structure promote long linear chain formation and interlayer distances of the C-S-H during the pozzolana reaction. This allows the Al to enter into the calcium silicate hydrate structure [9,10,11]. According to Kalousek [12], Al enters into the calcium silicate hydrate structure and primarily substitutes for Si in the tetrahedral position. Haas [13] investigated the dissolution of different kinds of C-(A)-S-H from both theory and practice aspects. They found that the incorporation of aluminum particles into C-A-S-H resulted in a decrease in its dissolution. The maximum Al/Si ratio is around 0.3. Lothenbach et al. [14] have demonstrated that the maximum value of Al/Si is 0.33. This indicates that the incorporation of aluminum into the silicon chain can alter the structure of C-(A)-S-H, and that it has an upper limit. The MK is an active aluminosilicate and an excellent pozzolanic material among the aluminum-rich pozzolan materials. The reserves of MK are widely distributed and have broad application prospects. Therefore, it has high research value and practical significance.

The primary binding phases used in cement-based materials are typically C-(A)-S-H gels, which have a direct impact on the performance of concrete. Many C-(A)-S-H models attempt to explain the essential properties of C-(A)-S-H structures. Jennings [15] proposed a comprehensive model, in which the C-(A)-S-H gel is assumed to be a particle colloid composed of 4–5 nm particles, which aggregate into a fractal structure with two typical morphologies. The low-density (LD C-(A)-S-H) gel is assumed to have an average packing density of 0.64, and the high-density (HD C-(A)-S-H) gel) has a value of 0.75 [16]. Li et al. [5] observed that Al/Si enhanced the structural density while increasing the bulk density of C-(A)-S-H gels using transmission microscopy. Furthermore, the presence of aluminum was observed to increase the degree of order of the C-(A)-S-H particles, since they tend to form foil structures rather than remain as individual particles. These methods were used to indirectly evaluate the micro-mechanical properties of C-(A)-S-H gels until nanoindentation was applied to the cementitious materials. Constantinides et al. [17,18] found that statistical nanoindentation can evaluate the phases based on different micro-mechanical properties, which are then represented by a specific elastic or hardness per phase. According to Jennings [15], the elastic modulus of LD C-(A)-S-H and HD C-(A)-S-H gel was 21.7 ± 2.3 GPa and 29.4 ± 2.4 GPa, respectively. Additionally, Haijing et al. [19] recognized the practical significance of using statistical techniques in the field of civil engineering. The copula method is an effective tool used in recent years to resolve multivariate variable dependency issues such as the tail dependence of data and has the following advantages. First, there are no restrictions on the form of the marginal distribution of multiple variables for the copula function. Consequently, a marginal distribution can be chosen that is suitable for the data while establishing a consistent relationship between the dependent variable and the independent variable. Moreover, the copula method can establish the dependency structure as well as the marginal distribution among variables [20]. The current focus of research is primarily on the impact of aluminum-rich pozzolanic material on the structure of C-(A)-S-H gel. Furthermore, the pozzolanic effect and filling effect were mainly used to explain the improvement in the micro-mechanical properties of the C-(A)-S-H gel phase. Few studies have directly studied the effect of aluminum on the elastic properties of C-(A)-S-H gel under different curing conditions. Furthermore, it is rare to demonstrate the dependence relationship using statistical principles.

The application of high-temperature steam curing is prevalent in numerous projects. It is a highly effective method for curing concrete, with significant application in the construction of high-strength concrete components. By applying temperature and steam, Li et al. [21] demonstrated that a higher early intensity can be achieved in a shorter curing time. According to Tian [22], high-temperature curing could effectively densify the microstructure of FA-CM cement-based material. Cheng [23] found that calcium sulfate whiskers significantly improved the macroscopic mechanical properties of the cement binders by forming the C-(A)-S-H phase and deflecting the defect phase in the matrix under high-temperature curing. Therefore, it is necessary to conduct a thorough investigation to establish an empirical formula for curing age, MK replacement rate, and the compressive strength of MK-cement cement-based material under different curing conditions.

The reserves of MK are widely distributed and have broad application prospects. It has high research value and practical significance. However, the present research on metakaolin mainly focuses on its pozzolanic ash and filling effect. Little research has directly studied the impact of aluminum on the elastic properties of C-(A)-S-H gel under different curing conditions. Furthermore, it is rare to demonstrate the dependence relationship using statistical principles. The main objective of this study is to investigate the macro- and micro-mechanical properties of the MK-cement cementing system under standard and high-temperature steam curing conditions. The compressive strength, flexural strength, and elastic modulus of the C-(A)-S-H gel phase served as evaluation indicators for the macroscopic and microscopic mechanical properties. Specifically, a compressive strength and flexural strength test was used to determine the optimal replacement rate and curing time of MK under high-temperature steam curing conditions and standard curing conditions. Additionally, the ACI empirical formula was employed to demonstrate the functional relationship between the MK replacement rate, curing time, and compressive strength. The effect of the hydration degree and Al/Si ratio of the C-(A)-S-H phase on its micro-mechanical properties was determined using thermogravimetric testing and nanoindentation combined with an SEM-EDX test. Furthermore, the copula function demonstrated the dependence of Al/Si on the elastic modulus of the C-(A)-S-H phase.

## 2. Materials and Methods

### 2.1. Raw Materials

Ordinary Portland cement with 52.5 grade (Jidong Co. Ltd., Hohhot, China) complies with the Chinese national standard GB175–2007. In this study, the ISO-standard sand had the fineness modulus of 2.8, and a bulk density of 1560 kg/m^3^. Metakaolin (Sanxing Co. Ltd., Hohhot, China) had an average particle size of 1.52 µm and a surface area of 10,000 m^2^/kg based on the manufacturer’s data. Table 1 summarizes the chemical compositions of raw materials which were provided by the manufacturer. The superplasticizer was used in this research, with a water-reducing rate of 40%, and the test dosage was uniformly 0.1% of the mass of the cementitious material.

### 2.2. Preparation of the Samples

Four series of samples with MK concentrations of 0%, 10%, 20%, and 30%, respectively, were prepared and designated as CM00, CM10, CM20, and CM30, respectively. Steam curing at high temperature and ordinary curing at room temperature are marked by the letters “H” and “S”. The figure on the end side is the curing time. For instance, CMH103 express that the MK substitution ratio is 10% metakaolin and was cured at a high temperature for 3 days. Each sample had water–binder ratio of 0.18 and a sand–binder ratio of 2. The mixed proportions are listed in Table 2.

Binder and sand were mixed at a low speed for 2 min during sample preparation. Following that, water was injected at a high pace for 5 min. Finally, the mixed slurry was cast into dimensions of 40 mm × 40 mm × 160 mm for a compressive strength and flexural strength test. After 24 h, the specimens were demolded and set in a high temperature box for 1, 3, and 6 days respectively. The curing temperature was 70 °C. The standard curing samples were stored at a temperature of 20 °C for 1, 3, 7, 14, and 28 days. All samples had a relative humidity of over 90%. Additionally, to carry out nanoindentation and SEM-EDS tests, another group of samples with the same combination and curing methods without sand were set. When the curing time was reached, 20-mm circular discs with a diameter of 35 mm were sliced from the middle of the prism. The discs and crumbs were immersed in isopropanol for 1 day and then vacuum-dried for SEM-EDS and nanoindentation tests.

### 2.3. Test Methods

**Figure 1:**
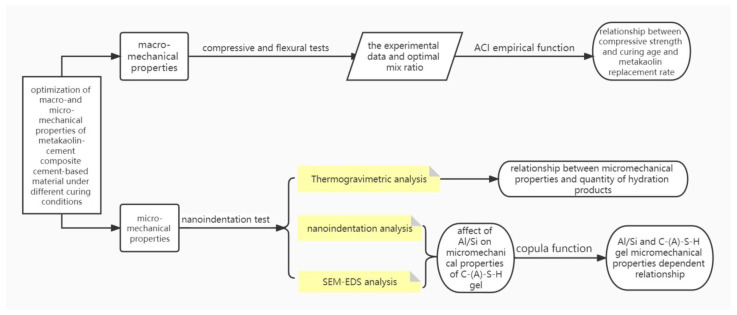
The research content and test procedure.

The research content and test procedure are shown in the Figure 1.

The flexural test refers to the Chinese national standard GBT 17671-2021. The specific process is as follows. The specimen’s sizes are 40 × 40 × 160 mm when they reach test age, the center loading method is used to measure the specimen’s flexural strength, where three samples are examined and their arithmetic averages are calculated. The calculation formula is as follows:

(1) $$R_{f}=1.5F_{f}L/b^{3}$$

where: $R_{f}$: flexural strength, MPa; $F_{f}$: maximum load at fracture failure, N; b: the length of the square section, b=40mm; L: distance between the center of supporting cylinder, L=100mm.

After the flexural test is completed, the compressive test is carried out on the two sections, in which the stress area is 40 × 40 mm. Six specimens are examined, and their arithmetic averages are calculated. The calculation formula is as follows:

(2) $$R_{c}=F_{c}/b^{2}$$

where, $R_{c}$:compressive strength, MPa; $F_{c}$: maximum load at crush, N; b: the length of the square section, b=40mm.

Using the Mettler–Toledo simultaneous thermal analysis apparatus (TGA/SDTA 851), the crumbs were pulverized to pass through a 45-µm filter for the TG/DTG tests. The samples were heated in an N_2_ environment from 5 to 1000 °C at a rate of 10 °C/min in an alumina crucible.

Nanoindentation tests were performed using the Ti950 Triboindenter nanoindenter. The load control mode was used in which the loading unloading cycle test was carried out for each measuring point in succession. The specific process, according to Reference [16]. In the nanoindentation test, a 7 × 7 indentation matrix was set with a spacing of 8 µm in each sample matrix. According to the Oliver–Pharr principle [17], the indentation modulus (E) at each measuring point is calculated as Equation (3):

(3) $$\frac{1}{E_{r}}=\frac{1-v^{2}}{E}+\frac{1-v^{2}}{E_{i}}$$

where: E: Elastic modulus of cement-based materials; v: Poisson ratio; $E_{i}$: indenter elastic modulus, GPa; for diamond indenters, $E_{i}=1141\mathrm{GPa}$, $v_{i}=0.07$; for cementitious materials v=0.25; studies have shown that $E_{r}$ of cementitious materials meet Gaussian distribution [24].

(4) $$f_{j}=\frac{N_{j}}{N}\;\;\sum_{j=1}^{m}N_{j}=N$$

(5) $$\mu_{j}^{\alpha }=\frac{1}{N_{j}}\sum_{n=1}^{N_{j}}x_{i}^{\alpha }\;(\sigma_{i}^{\alpha }{)}^{2}=\frac{1}{N_{j}-1}\sum_{n=1}^{N_{j}}(x_{i}^{\alpha }-\mu_{j}^{\alpha }{)}^{2}$$

where $x_{i}^{\alpha }$ represents one such mechanical property (α=(M, H) from a single indentation test, and $f_{j},\;\mu_{j}^{\alpha }$, and $\sigma_{j}^{\alpha }$ stand for volume fraction, mean, and standard deviation of mechanically distinct phases, respectively. Also $N_{j}$ denotes the number of indentation on material phase j and N is the total number of indentations test. the core purpose of any deconvolution method is to estimate five unknowns, {$\mu_{j}^{M}$,$\sigma_{j}^{M}$,$\mu_{j}^{H}$, $\sigma_{j}^{H}$,$f_{j}$} per phase j [13,16].

Scanning electron microscopy (JEOL JSM6500) and energy dispersive spectroscopy (EDS) were used to analyze the microstructural and chemical constituents of C-(A)-S-H phase. Spot analyses were performed just adjacent to the indentation imprint to avoid errors introduced by the local surface roughness. Analyses were conducted with an accelerating voltage of 15 KV, and a 10 mm working distance to match the range of SEM-EDS analysis on C-(A)-S-H gel with the nanoindentation results [24].

## 3. Results and Discussion

### 3.1. Analysis of the Macro-Mechanical Properties of MK-Cement Cementitious System

#### 3.1.1. Compressive Strength

**Figure 2:**
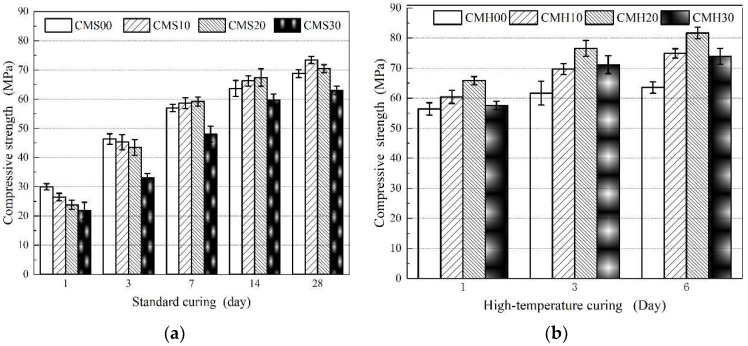
Compressive strength development of the composite cement-based material under standard curing (**a**) and high temperature curing (**b**).

The MK influences the properties of cementitious materials and can significantly improve the macro-mechanical properties of composite cement-based materials. Moreover, the optimal replacement rate of MK is crucial for its application and further research. The compressive strength of the samples under standard curing is depicted in Figure 2a. Within the first three days, MK lowers the compressive strength of the samples. The higher the MK content, the lower the early strength, which indicates MK’s low pozzolanic ash reaction in the early stage of hydration. After seven days, the compressive strength of the samples, which were mixed with MK, started to exceed that of the reference group. The 28-day compressive strength was systematically stronger than the reference sample, which increased with the increase in the MK replacement rate before it was set at 10%. Moreover, the compressive strength tended to decrease as the MK increased. After 28 days, the CMS1028 sample obtained the maximum compressive strength, and the compressive strength reached 73.45 MPa. Its compressive strength was 6.35%, 4.06%, and 14.25% higher than that of CMS0028, CMS2028, and CMS3028, respectively. Thus, it is reported that MK can effectively improve the macroscopic mechanical properties of cement-based materials and the optimal replacement rate of MK is 10% under standard curing conditions.

Another finding, presented in Figure 2b, is that the compressive strength of samples increased rapidly under high-temperature steam curing. As shown in Figure 2b, on day 1, the compressive strength of both CMH101 and CMH201 is comparable to that of CMS2014. Furthermore, CMH203, at a later age, attained a maximum compressive strength of 76.57 MPa, which was 1.09, 1.04 and 1.08 times higher than that of CMH103, CMS1028, and CMS2028, respectively. Although CMH206 attained the maximum compressive strength after six days, the growth rate was only 6.25% as compared with CMH203. Meanwhile, the compressive strengths of CMH006, CMH306, and CMH306 increased by just 2.93%, 6.93%, and 3.69%, respectively. This indicates that within 3 days, the high-temperature curing can significantly improve the compressive strength of the samples. Therefore, the findings reveal that high-temperature curing significantly enhances the MK replacement rate and compressive strength, and optimizes the curing time. The optimal MK replacement rate is 20%, and the optimized curing time is three days.

#### 3.1.2. Flexural Strength

**Figure 3:**
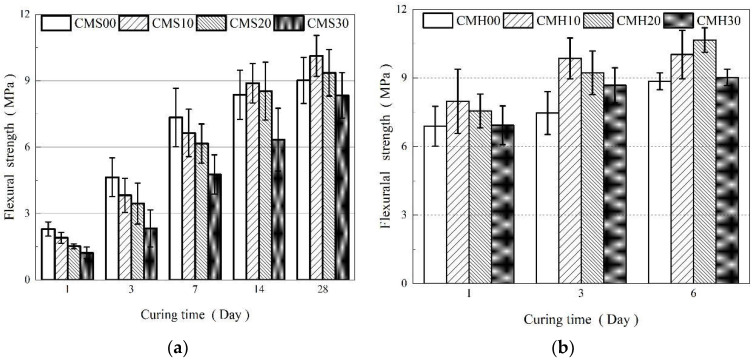
Flexural strength development of the composite cement-based material under standard curing (**a**) and high temperature curing (**b**).

Figure 3 displays the flexural strength of each sample. In Figure 3a, even though the variation trend in the flexural strength with varying MK replacement rates and curing ages is similar to that in the compressive strength, there are certain differences. MK improves the flexural strength in the standard curing series, but this effect is not apparent. CMS1028 attains the maximum flexural strength, which is only 12.19% higher than that of the control group. Under high temperature steam curing, the flexural strength exhibits an obvious improvement, as shown in Figure 3b. The flexural strengths of CMH106 and CMH206 were increased by 13.21% and 16.89%, respectively, in comparison to the reference group, and the best flexural strength of CMH206 was 10.67 MPa. Thus, this conclusion verifies that high-temperature curing effectively improves the MK replacement rate and improves the macroscopic mechanical properties of the composite cement-based materials.

The improvement in the macroscopic mechanical properties of cement-based materials is mainly due to the optimization of microstructure, such as the improvement in micro-mechanical properties and the reduction in the defects and cracks in the matrix. In this paper, metakaolin significantly promotes cement-based materials’ compressive strength and flexural strength. In view of this, the optimization of metakaolin under different curing conditions on the optimization of the microstructure of composite cement-based materials has high research value.

#### 3.1.3. ACI Prediction Model for Compressive Strength of MK-Cement Cementitious Materials

Engineering practices benefit significantly from establishing the relationship between MK replacement rate, curing age, and compressive strength under high-temperature curing and standard curing conditions. The ACI empirical model can well describe the development of the compressive strength of MK-cement cementitious materials [25]. In this paper, the regression analysis of the data in Figure 2a,b shows that it has good consistency with the ACI empirical model, and the correlation coefficient are all above 0.978. The model is as follows in Equations (6) and (7):

(6) $$f_{CUS}(t)=\frac{t}{a+bt}f_{CUH.6}$$

(7) $$f_{CUS}(t)=\frac{t}{c+dt}f_{CUS.28}$$

where: t: curing age, day; a, b, c, d: regression coefficient, a+6b=6, c+28d=28: $f_{CUH.6}\;f_{CUS.28}$: cement cementitious materials under high-temperature steam curing 6 days and standard curing 28 days, respectively, MPa.

**Figure 4:**
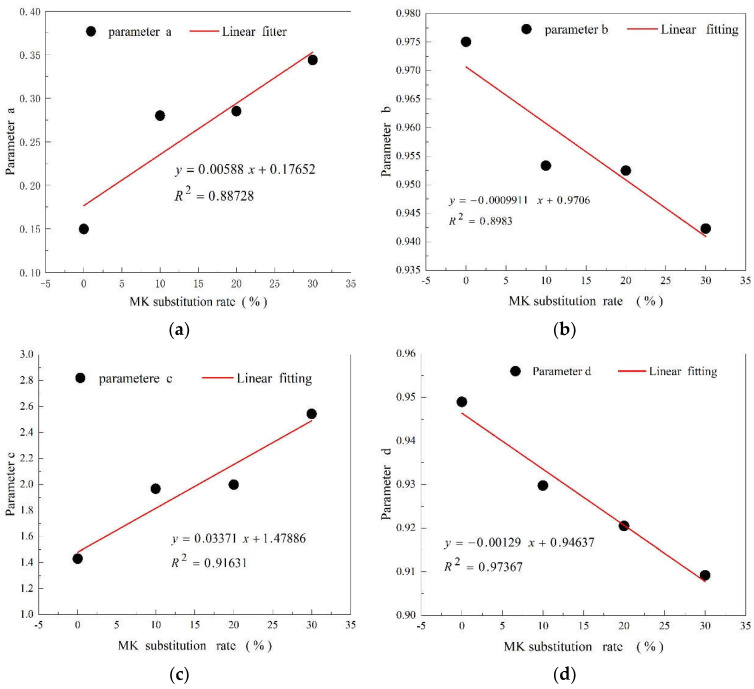
Relation among coefficients (**a**–**d**) and replacement rate of MK.

Accordingly, this paper constructs functions of different MK substitution rates under different curing conditions and regression coefficients a, b, c, and d, respectively. the regression coefficients a, b, c and d of each curve in Figure 4a–d are found to have a good linear relationship with the MK replacement rate, and the correlation coefficients are all above 0.88. This shows that the ACI prediction model can better predict the effect of the MK replacement rate and curing time on compressive strength. The relevant equations are as follows:

(8) $$\begin{array}{c} a=0.00588MK+0.17652\\ b=-0.0009911MK+0.9706 \end{array}$$

(9) $$\begin{array}{c} c=0.03371MK+1.47886\\ d=-0.00129MK+0.94637 \end{array}$$

The regression coefficient a, b, c and d are brought into prediction model Equations (3) and (4) and the prediction model of compressive strength under high-temperature and standard curing conditions are obtained as Equations (10) and (11):

(10) $$f_{cu}(t)=\frac{t}{0.00588MK+0.17652+t(-0.0009911MK+0.9706)}f_{cu,6}$$

(11) $$f_{cu}(t)=\frac{t}{0.03371MK+1.47886+t(-0.00129MK+0.94637)}f_{cu.28}$$

**Figure 5:**
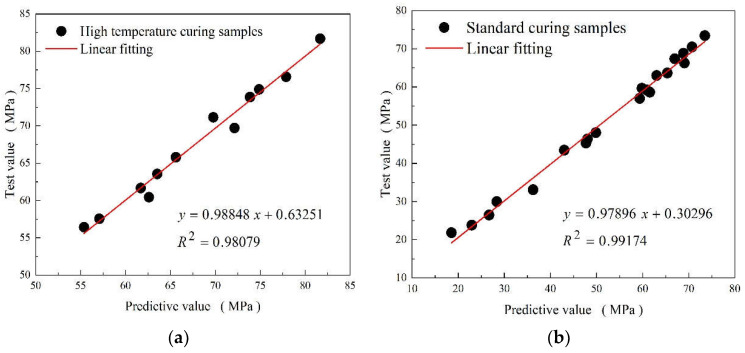
Relation between the Predictive value and test value of composite cementitious (**a**) under high temperature curing and (**b**) standard curing conditions.

There is a clear linear positive relationship between the predicted value of the ACI prediction model for the compressive strength of MK-cement cementing systems under high-temperature steam and standard curing and the measured value. In Figure 5a,b, the scatter data are evenly distributed near the line with a slope close to 1, indicating the high accuracy of the model and its ability to quickly calculate the compressive strengths of MK-cement cementitious materials with different MK replacement rates and curing ages, which can be used in practical projects or related tests.

### 3.2. Analysis of the Micro-Mechanical Properties of MK-Cement Cementitious System

#### 3.2.1. Nanoindentation Testing

**Figure 6:**
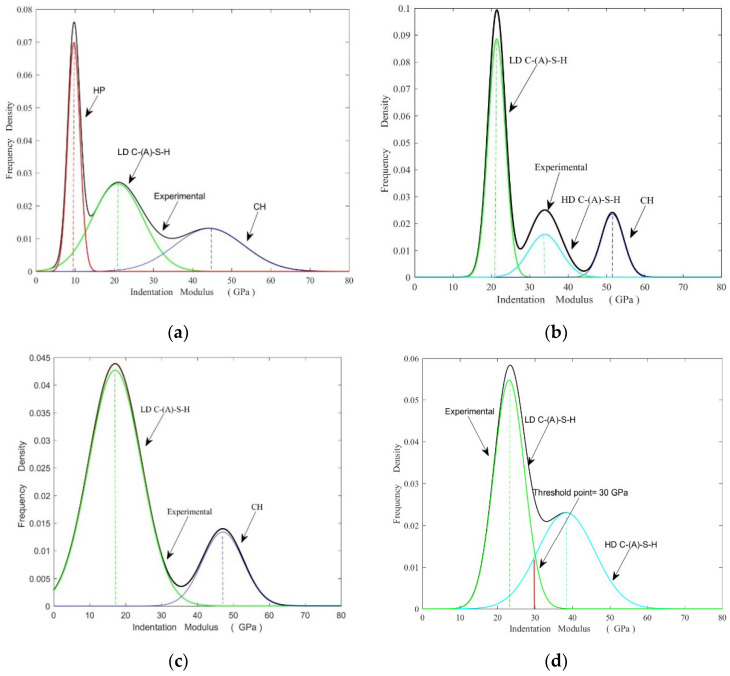
Nanoindentation point Gaussian fitting results of sample (**a**) CMH006, (**b**) CMS1028, (**c**) CMS0028, and CMH206 (**d**).

C-(A)-S-H (calcium aluminosilicate hydrate) gels, particularly the HD C-(A)-S-H (high density- calcium aluminosilicate hydrate) gel, improve density, reduce unevenness in the matrix and effectively improve the macroscopic performances of cementitious composite materials [26]. Therefore, understanding the micro-mechanical properties of C-(A)-S-H gel is crucial for practical applications. The statistical nanoindentation technique is used to evaluate parameters such as elastic modulus and hardness of C-(A)-S-H gels. According to the corresponding literature [16,17,18], the elastic range of each phase in cement-based materials is HP (defect phase) (0–12 GPa), LD C-(A)-S-H (low density- calcium aluminosilicate hydrate) (13–25 GPa), HD C-(A)-S-H (26–38 GPa), and CH (>38 GPa). In this study, Gaussian multimodal fitting was used to analyze each sample’s nanoindentation elastic modulus values under high-temperature steam and standard curing conditions and draw a probability density map. As shown in Figure 6a–d, there have multiple repetitions along the curve, but the peaks are identifiable. In particular, CMH006 fitted a large number of defect phases. However, CMH206 only fit the C-(A)-S-H gel phase, in which the HD C-(A)-S-H gel content was as high as 44%. This shows that MK and curing methods significantly affect the micro-mechanical properties and phase composition of the composite cementing system.

**Figure 7:**
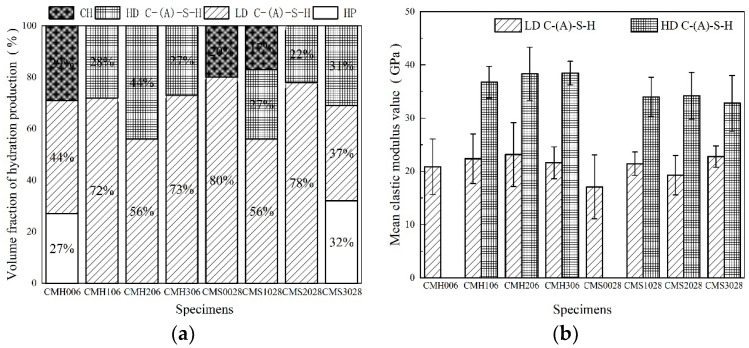
The volume fraction of samples (**a**) and Gaussian fitting average elastic modulus value (**b**) of C-(A)-S-H gel.

Figure 7 shows the variation of average elastic modulus of C-(A)-S-H gel and hydration product content under high temperature steam and standard curing conditions. As shown in Figure 7a, the defect phase content and the CH phase decrease gradually as the MK substitution ratio increases. In the high-temperature curing series, only CMH006 fit the defect phases. This indicates that MK could consume CH crystal, increase te C-(A)-S-H gel phase, and repair matrix defect, and then increase the matrix compactness. Moreover, it is noteworthy that CMS3028 fits the defect phase, which indicates that a high MK replacement rate lowers its degree of hydration. The specific reasons are as follows: large amounts of unreacted MK particles of small sizes adhere to the surface of the cement clinker particles, hindering their hydration. Moreover, the layered structure of MK and the adsorption of a large amount of free water in the mixed slurry impede the hydration of cement clinkers. Furthermore, a large amount of cement clinker is replaced by MK, reducing the pH value of the mixed slurry and the amount of cement clinker, which is detrimental to cement hydration and the pozzolanic effect of MK [27]. However, CMH306 also has the MK replacement rate of CMS3028, but no defect phase is fitted, as shown in Figure 7a. This is because the pozzolanic activity of MK is fully stimulated under high-temperature curing and then repairs the defect in the matrix. Another noteworthy aspect is that MK can effectively lower the content of CH crystals in the matrix. As depicted in Figure 6a, only CMH003, CMS0028, and CMS1028 fit the CH crystals. CH crystal structure is coarse and particular orientation in the matrix. Despite having a large elastic modulus, it cracks easily and its uneven distribution causes the microstructure to become loose and finally diminishes the macroscopic mechanical properties.

The average elastic modulus of C-(A)-S-H gel is shown in Figure 7b. The elastic modulus value of the C-(A)-S-H gel phase, particularly the HD C-(A)-S-H phase, can be improved by incorporating MK. In sample CMH206, the percentage of HD C-(A)-S-H is 44%, and the average elastic modulus is 38.33 GPa. The average elastic modulus of the HD C-(A)-S-H phase in CMS1028, CMS2028, and CMH106 was lower by 4.15%, 11.43%, and 10.82%, respectively, compared with CMH206. The conclusion is that MK can effectively promote the average elastic modulus of the gel phase, which is more evident under high-temperature steam curing conditions. This phenomenon is evident in be explained below.

Thus, MK can repair matrix defects, reduce the CH crystal content, and improve the elastic modulus of the C-(A)-S-H gel phase, which is evident under high-temperature curing conditions. Furthermore, MK optimized the micro-mechanical properties of the composite cementitious system at the microscopic level. This optimization mechanism will be analyzed based on two aspects: the degree of hydration of the composite cementitious system and the Al/Si of the C-(A)-S-H phase.

#### 3.2.2. Analysis of Hydration Mechanism

**Figure 8:**
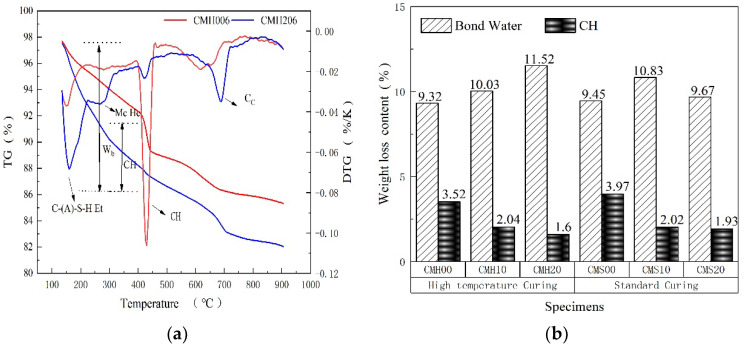
(**a**) Thermogravimetric analyses curve (TG and DTG) and (**b**) hydration products analysis.

Hydration, defined as the mass conversion of different phases in a composite cementitious system, is related to the properties of pozzolanic materials. The properties of pozzolanic materials have a significant effect on their macro-and micro-mechanical properties [28]. A novel C-(A)-S-H gel is formed when aluminum-rich pozzolan materials, such as MK, react with the CH crystal during cement clinker hydration. Additionally, a portion of aluminum in MK will increase the alumino–silicate hydrates by consuming a certain amount of CaCO_3_ in the pozzolanic reaction. As shown in Figure 8a, sample CMH006 exhibits a weak signal for Hc and Mc, which is more evident in CMH206. Therefore, aluminum-rich pozzolan materials can increase the type of hydration products. However, C-(A)-S-H remains the main hydration product. One of the frequently used ways of determining the hydration degree of a composite cementitious system is the formation of non-evaporable water (w), the information of which is based on prior literature [26]. Suraneni et al. [29] observed that the weight loss temperature range of bound water and CH crystals is 40–500 °C and 400–500 °C, respectively.

To determine the impact of MK on the hydration of composite cement-based material, the bound water content and MK content were measured under standard and high-temperature steam curing conditions. The test for TG/DTG is shown in Figure 8b. In the composite cementitious systems, MK promotes the bound water content, which can offset the effect of dilution. In comparison with the control group, the combined water content of CMS1028 and CMS2028 for the standard curing series increased by 5.68% and 2.33%. The growth rates in the high-temperature steam curing series are 4.06% and 12.52%, respectively. The bound water content of each sample in the high-temperature curing series is higher than that in the standard curing sample corresponding to the replacement rate. This further suggests that the pozzolanic effect of MK can be effectively promoted by high-temperature steam curing. The number of C-(A)-S-H phases per unit space in the matrix increases as the number of hydration products increases. Moreover, the compactness of the matrix is increased, leading to an increase in the elastic modulus of C-(A)-S-H gel.

Additionally, MK effectively reduces the CH crystals in the matrix. The CH crystal contents were computed as a significant mass drop in the TG curve at temperatures between 400 °C and 500 °C. In Figure 8b, the content of CH crystals is only 1.6% for the CMH206. However, only a few samples fit the CH phase in the nanoindentation test. This implies that the pozzolanic reaction of MK decomposes the bulky CH crystals into small particles, which aids in increasing the uniformity and reducing the defect phase in the matrix. Additionally, an intimate nanocomposite of C-(A)-S-H and nanoscale CH resides in the gel pores of C-(A)-S-H, which increases the elastic modulus of the C-(A)-S-H phase [24]. This also explains why MK increases the average elastic modulus of the C-(A)-S-H phase during high-temperature steam in Section 3.2.

#### 3.2.3. Effect of Al/Si on Micro-Mechanical Properties of C-(A)-S-H Gel

**Figure 9:**
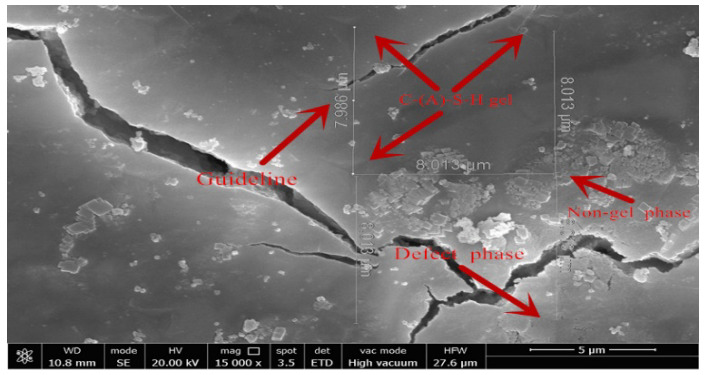
Nanoindentation morphology of sample CMH106 by SEM.

Chen et al. [24] verified that the chemical information of C-(A)-S-H obtained from SEM-EDS was directly related to the micro-mechanical properties obtained by nanoindentation in an attempt to further investigate the enhancement effect of aluminum elements on the micro-mechanical properties of the C-(A)-S-H phase. In this paper, SEM-EDS was performed in a point-by-point mode at each nanoindentation. Due to the interaction volume of electrons with the specimen and the intergrowth of hydrates, the detected X-rays of one spot typically consist of a signal of a mix of two or more phases [30,31]. In this paper, the element ratio plots of Al/Si were used to characterize the formation of a phase. Moreover, the influence of defect phases, CH phases, and unhydrated particles must be eliminated, as shown in Figure 9. The indentation points with an elastic modulus less than 12 GPa and greater than 40 GPa were eliminated. The theshold point for defect phase and LD C-(A)-S-H was 12 GPa, and LD C-(A)-S-H and HD C-(A)-S-H gels were distinguished by the threshold (30 GPa) of sample CMH206 in Figure 5d.

Table 3 presents the Ca/Si and Al/Si of the samples. The Ca/Si of the samples progressively decreased with an increase in the MK replacement rate, while the Al/Si improved gradually. The Al/Si in the samples CMH306 and CMS3028 exceeds 0.43. According to Richardson [32], the maximum Al/Si in the silicon chain structure is around 0.3, which indicates that a large amount of unreacted MK impacts the EDX results. This finding is consistent with TG/DTG test results in 3.2.2. Furthermore, the quantity of HD C-(A)-S-H gel increased significantly with the increase in Al/Si in C-(A)-S-H gel among the samples that underwent the same curing. For CMH206 and CMH306, the HD C-(A)-S-H reached 44% and 32%, respectively in Figure 6a. Furthermore, the average Al/Si of the HD C-(A)-S-H gel phase was higher than that of the LD C-(A)-S-H phase. The conclusion is that Al/Si has a remarkable promotion for improving the micro-mechanical properties of the C-(A)-S-H gel phase. This is due to the formation of C-(A)-S-H gels by the accumulation of numerous C-(A)-S-H particles. As the MK substitution rate increases, more Al elements enter the silicon chain, alter the structure of the silicon chain, and increase the packing density of the C-(A)-S-H gel. Furthermore, Si-Al-O is a ternary space network structure with a better degree of polymerization than the Si-O binary plane structure [16,33]. The gelling structures complement each other, which can improve the mechanical properties of the C-(A)-S-H gel phase.

#### 3.2.4. The Dependency between Al/Si and Micro-Mechanical Properties of C-(A)-S-H Gel

In recent years, the copula function has been used as an effective tool to characterize the dependence structure among random variables. This function serves as a connection function between the joint distribution and the marginal distribution function. The fundamental idea is to construct the marginal distribution function and the copula function independently [34]. Under the framework of copula theory, the joint distribution model of any group of variables can be established, and neither the marginal distribution function type nor the related structure type is restricted by the type. Because of its unparalleled flexibility and applicability in establishing the joint distribution model of variables, copula theory has been extensively used in civil engineering and other fields.

To further verify the promoting effect of Al/Si on the micro-mechanical properties of the C-(A)-S-H phase, the correlation between the elastic modulus values of the nanoindentation points and the corresponding Al/Si in CMH206 is demonstrated in this paper using the copula function.

In the copula function, it is assumed that $X_{1},X_{2},\ldots ,X_{n}$ are the random variables and their marginal cumulative distribution functions (CDFs) are $F_{1}(x_{1}),F_{2}(x_{2}),\ldots ,F_{n}(x_{n})$.

According to Sklar’s theorem, if $H(x_{1},x_{2},\ldots ,x_{n})$ is a joint distribution function of $X_{1},X_{2},\ldots ,X_{n}$ with marginal CDFs $F_{1}(x_{1}),F_{2}(x_{2}),\ldots ,F_{n}(x_{n})$, then there exists a copula function *C* such that $H(x_{1},x_{2},\ldots ,x_{n})=C(F_{1}(x_{1}),F_{2}(x_{2}),\ldots ,F_{n}(x_{n}))$. Let $u_{i}=F_{i}(x_{i})$ the joint probability density function (pdf) $h(x_{1},x_{2},\ldots ,x_{n})$ of $X_{1},X_{2},\ldots ,X_{n}$ is $h(x_{1},x_{2},\ldots ,x_{n})=c(u_{1},u_{2},\ldots ,u_{n})\prod_{i=1}^{n}f_{i}(x_{i})$, where $c(u_{1},u_{2},\ldots ,u_{n})=\frac{\partial^{n}C(u_{1},u_{2},\ldots ,u_{n})}{\partial u_{1}\partial u_{2}\ldots \partial u_{n}}$ and $f_{i}(x_{i})$ is the pdf of $X_{i},\;i=1,2,\ldots ,n$.

The elliptic copula function is the more commonly used single-parameter copula function family, such as Gaussian and *t* copula, and Archimedean copulas, such as Clayton, Gumbel, and Frank Copula. Each copula function has intrinsic tail features. Therefore, the selection of copula functions is crucial. The appropriate copula function is chosen according to the square of the Euclidean distance $d^{2}$ between the copula function and the empirical copula function. The smaller the $d^{2}$, the better the fit of the copula function [35].

In this paper, we assumed that the Al/Si of nanoindentation point is the random variable *X* and elasticity modulus value of C-(A)-S-H gel is the random variable *Y*, and both *X* and *Y* obey the Gamma distribution, and denote as $X\sim G(\alpha_{1},\beta_{1})$$Y\sim G(\alpha_{2},\beta_{2})$. Its probability density function is Equation (12).

(12) $$f(x;\alpha_{1},\beta_{1})=\frac{\beta_{1}{}^{\alpha_{1}}}{\Gamma (\alpha_{1})}x^{\alpha_{1}-1}e^{-\beta_{1}x};\;g(y;\alpha_{2},\beta_{2})=\frac{\beta_{2}{}^{\alpha_{2}}}{\Gamma (\alpha_{2})}y^{\alpha_{2}-1}e^{-\beta_{2}y};$$

Additionally, the dependency between Al/Si and micro-mechanical properties of C-(A)-S-H gel established by Gumbel copula and Clayton copula.

Based on the maximum likelihood method and the inference function for marginal IFM method, which is a new parameter estimation method, the Gamma distribution and the parameter estimates in the copula function are derived. The goal of this approach is to obtain the parameter estimates of the marginal distribution and the parameter estimates of the joint distribution function, respectively. Thus, based on the sample observations of *X* and *Y*, the likelihood function of the group of samples is obtained as Equations (13) and (14):

(13) $$L_{1}(\alpha_{1},\beta_{1};X)={\left(\frac{\beta_{1}{}^{\alpha_{1}}}{\Gamma (\alpha_{1})}\right)}^{n}\prod_{i=1}^{n}x^{\alpha_{1}-1}\exp(-\beta_{1}x)$$

(14) $$L_{2}(\alpha_{2},\beta_{2};Y)={\left(\frac{\beta_{2}{}^{\alpha_{2}}}{\Gamma (\alpha_{2})}\right)}^{n}\prod_{i=1}^{n}y^{\alpha_{2}-1}\exp(-\beta_{2}y)$$

According to the above likelihood Functions (13) and (14), the maximum likelihood estimates of parameters $\alpha_{1},\;\beta_{1},\;\alpha_{2},\;\beta_{2}$ are obtained by numerical method and represented as ${\hat{\alpha }}_{1},\;{\hat{\beta }}_{1},\;{\hat{\alpha }}_{2},\;{\hat{\beta }}_{2}$.

Then, according to parameter estimate value of the marginal distribution, we obtained the likelihood function of the joint distribution function Equation (15):

(15) $$L_{4}(\theta ;X,Y,{\hat{\alpha }}_{1},{\hat{\beta }}_{1},{\hat{\alpha }}_{2},{\hat{\beta }}_{2})=\prod_{i=1}^{n}c(F(x_{i};{\hat{\alpha }}_{1},{\hat{\beta }}_{1}),G(y_{i},{\hat{\alpha }}_{2},{\hat{\beta }}_{2}))$$

ML (maximum likelihood estimate) methods were used to obtain the parameter values of the margin distribution, and the K-S test was applied to explain the fitting data of the margin distribution. The conclusion is shown in Table 4.

The estimates of dependence parameters were calculated based on the parameter estimations of the margin distribution. According to the above model parameter estimates and dependence parameter estimates, the distance between the selected Copula function and the empirical copula function was calculated. The most appropriate Copula function is that with the smallest distance. As presented in Table 5, the Gumbel function is the most appropriate Copula function to describe the Al/Si dependencies of the C-(A)-S-H gel and its elastic modulus value. The dependency parameter was estimated to be 5.320965, indicating a strong correlation between Al/Si of C-(A)-S-H gel and its elastic modulus.

## 4. Conclusions

In this paper, the macro-and micro-mechanical properties of MK-cement composite cementitious materials were examined based on compressive strength, flexural strength, and nanoindentation under standard and high-temperature steam curing conditions. The ACI empirical formula was established. Furthermore, the effect of the hydration degree and Al/Si on the micro-mechanical properties of the C-(A)-S-H phase was explored, and their dependence was demonstrated by the copula function. The specific conclusions are as follows:

1. Under the high-temperature curing conditions, the compressive strength and flexural strength of the metakaolin-cement composite cementitious materials were effectively enhanced within a short time. Moreover, the optimal replacement rate of MK was increased from 10% to 20%. Finally, under standard and high-temperature steam curing circumstances, the ACI empirical model for compressive strength, curing time, and MK replacement rate was established.
2. The pozzolanic activity of MK is adequately stimulated under high-temperature curing conditions, which increases the types and quantities of hydration products and reduces the content of CH crystals in the matrix. Furthermore, MK increases the content of the C-(A)-S-H phase, especially the HD C-(A)-S-H phase, and enhances their average elastic modulus values. This may lead to a decrease in the defects and formation of dense microstructures.
3. MK can increase the Al/Si and decrease the Ca/Si ratios of the C-(A)-S-H phase. The increase in Al/Si in the C-(A)-S-H phase contributes to an increase in its elastic modulus, bulk density, and HD C-(A)-S-H phase content. This effect is more pronounced under high-temperature curing conditions.
4. The Gumbel copula function demonstrates the dependence of the elastic modulus values of the C-(A)-S-H gel phases and Al/Si. To be specific, the classical maximum likelihood method was combined with the IFM method to obtain the parameter estimates of the margin distribution, and the corresponding K-S test was performed. By solving the Euclidean distance between alternative copula and empirical copula, it has been verified that the use of Gumbel copula to describe the dependence between the C-(A)-S-H gel and Al/Si is reasonable, and the estimated value of the Gumbel copula parameter of 5.320965 confirms strong dependence.

In the future, we will explore the upper limit of aluminum incorporation into the C-(A)-S-H structure under different curing conditions. In addition, the influence of aluminum on the C-(A)-S-H structure and its micro-mechanical properties were further studied.

## Figures and Tables

**Table 1:** The chemical composition of raw materials kg/m^3^.

Raw Material	CaO	SiO_2_	MgO	AL_2_O_3_	Fe_2_O_3_	Na_2_O	LOI
Cement	62.68	21.28	3.61	4.82	4.34	0.41	2.86
Metakaolin	0.2	50	0.09	46	2.6	0.4	0.71

**Table 2:** Mixed proportion for mortar kg/m^3^.

Sample	PC	MK	Sand	Water
CMH00, CMS00	1.00	-	2.00	0.18
CMH10, CMS10	0.90	0.10	2.00	0.18
CMH20, CMS20	0.80	0.2	2.00	0.18
CMH30, CMS30	0.70	0.30	2.00	0.18

**Table 3:** Mean Ca/Si and Al/Si atom ratios obtained for C-(A)-S-H using SEM-EDX (A.M. = arithmetical mean; S.D. = standard deviation; N. = number of analyses).

Code.	LD C-(A)-S-H					HD C-(A)-S-H				
	Al/Si		Ca/Si			Al/Si		Ca/Si		
	A.M.	S.D.	A.M.	S.D.	N.	A.M.	S.D.	A.M.	S.D.	N.
CMH106	0.23	0.05	1.97	0.53	22	0.29	0.02	1.93	0.24	11
CMH206	0.29	0.07	1.79	0.42	26	0.32	0.03	1.83	0.39	15
CMH306	0.45	0.06	1.48	0.21	22	0.55	0.02	1.52	0.19	10
CMS1028	0.25	0.04	1.93	0.65	29	0.29	0.03	1.95	0.37	10
CMS2028	0.28	0.03	1.77	0.37	20	0.32	0.05	1.78	0.12	9
CMS3028	0.42	0.06	1.51	0.33	16	0.52	0.09	1.49	0.29	6

**Table 4:** Estimations for K-S distances and *p*-values of margin distribution parameters.

Data	Parameter Estimates		K-S Distances	*p*-Value
X	$\alpha_{1}$	43.480387	0.17199	0.4015
	$\beta_{1}$	141.77812		
Y	$\alpha_{2}$	17.0005357	0.12514	0.7456
	$\beta_{2}$	0.5458059		

**Table 5:** Dependency parameter and Euclidean distance *d*^2^ between the copula function and the empirical copula based on M.L.

Copula	Parameter Estimates	*d* ^2^
Gumbel	5.320965	0.01335448
Claycon	4.626953	0.0208742

## Data Availability

Not applicable.
